# Ongoing and completed clinical trials on prostate cancer screening using MRI

**DOI:** 10.1007/s00330-026-12533-4

**Published:** 2026-04-16

**Authors:** Jonas Wallström, Fredrik Jäderling, Fredrik Langkilde, Rebecka Arnsrud Godtman, Ola Bratt, Erik Thimansson

**Affiliations:** 1 Department of Radiology, Institute of Clinical Sciences, Sahlgrenska Academy, University of Gothenburg, Gothenburg, Sweden; 2 Department of Radiology, Sahlgrenska University Hospital, Gothenburg, Sweden; 3 Department of Molecular Medicine and Surgery (MMK), Karolinska Institutet, Solna, Sweden; 4 Department of Radiology, Capio S:t Görans Hospital, Stockholm, Sweden; 5 Department of Urology, Institute of Clinical Sciences, Sahlgrenska Academy, University of Gothenburg, Gothenburg, Sweden; 6 Department of Urology, Sahlgrenska University Hospital, Gothenburg, Sweden; 7 Diagnostic Radiology, Department of Translational Medicine, Lund University, Malmö, Sweden; 8 Department of Radiology, Helsingborg Hospital, Helsingborg, Sweden

**Keywords:** Magnetic resonance imaging, Mass screening, Prostatic neoplasms

## Abstract

**Abstract:**

Before the introduction of prostate MRI, prostate cancer screening relied on prostate-specific antigen (PSA) testing followed by a systematic biopsy. Although this approach reduced prostate cancer mortality in large, randomized screening trials, its net benefit was questionable because of substantial overdiagnosis. In response, more precise screening tools have been developed. Diagnostic studies demonstrate that MRI has a high sensitivity for clinically significant prostate cancer and a low sensitivity for low-grade cancer, making it well-suited as a triage before biopsy. Using MRI as the primary screening test increases detection of potentially clinically significant cancer, while sequential strategies—such as those used in the STHLM3-MRI trial—reduce the number of MRI scans, unnecessary biopsies and low-grade cancer diagnoses with maintained detection of clinically significant cancer. Several ongoing randomized trials now incorporate PSA-triggered MRI in repeated screening rounds. The Gothenburg 2 trial has reported that its MRI-targeted biopsy pathway halves low-grade cancer detection but misses a few clinically significant cancers. Proscreen evaluates a screening pathway with an ancillary kallikrein test to select men for MRI, PROBASE start age 45 years, and large-scale initiatives—such as organized prostate cancer testing (OPT) programs—evaluate real-world feasibility. Key challenges include optimizing MRI protocols, improving consistency in image interpretation, and reducing false-positive findings, particularly in younger men. Centralized reading, AI support, and refined risk stratification may enhance scalability. Accumulating evidence from ongoing trials and population-based programs suggests that MRI-based screening strategies improve the benefit-to-harm ratio in prostate cancer screening.

**Key Points:**

***Question*** *This special report summarizes the evidence from ongoing and completed clinical trials on prostate cancer screening using MRI.*

***Findings*** *Across diagnostic and randomized screening trials, MRI-based pathways reduce low-grade cancer detection and biopsy rates while maintaining detection of clinically significant prostate cancer.*

***Clinical relevance*** *MRI-based screening improves the benefit–harm balance by avoiding unnecessary biopsies and overdiagnosis while maintaining early detection of aggressive prostate cancer, enabling safer, more precise population-level screening for men.*

## Introduction

Before the era of magnetic resonance imaging (MRI), prostate cancer screening relied on prostate-specific antigen (PSA) testing followed by systematic biopsies [1]. If the PSA value exceeded a threshold, usually 3 ng/mL, the prostate was systematically sampled with 10–12 transrectal ultrasound (TRUS)-guided biopsy cores distributed throughout the gland. This “blind” approach led to both underdiagnosis of randomly missed important cancers and overdiagnosis due to accidentally sampling small, low-grade cancers with little or no potential to metastasize [2].

The European Randomized Study of Screening for Prostate Cancer (ERSPC) recently reported a 13% reduction in prostate cancer-specific mortality after 23 years of follow-up. This required inviting 456 men to screening and diagnosing 12 to prevent one prostate cancer death [3]. A separate analysis from the Gothenburg part of the ERSPC showed a 29% mortality reduction after 22 years, but still 9 men were diagnosed to prevent one death [4]. Consequently, population-based prostate cancer screening with PSA and systematic biopsies has been discouraged because the benefit of cancer mortality reduction has not been considered to outweigh the potential harms of overdiagnosis and overtreatment [5, 6]. In response, efforts have intensified to identify better screening methods, including additional blood tests, genetic markers, and imaging.

Among these, prostate MRI meets several requirements for a suitable test in screening [7]. With modern scanners, a noninvasive bi-parametric protocol—using single-plane T2-weighted and diffusion-weighted imaging sequences—can be performed in under 10 min [8], making it a fast, available, and acceptable test to most men [9]. In addition, the diagnostic profile of prostate MRI with a very high sensitivity for clinically significant prostate cancer and low sensitivity for low-grade cancer makes it ideal as a pre-biopsy filter [10–12]. In the clinical setting, pre-biopsy MRI with lesion-targeted biopsies has become standard care and is recommended by the EAU guidelines [13].

However, false-positive MRI findings, especially indeterminate (PI-RADS 3) lesions, are a limitation in a screening setting. Factors that may contribute to false positives include low image quality [14] and inter-reader variability, amplified by a lack of radiologists with screening expertise [15]. In addition, the current PI-RADS version was designed for clinical diagnosis, not population-level screening [16]. It may underperform in younger men with smaller, denser prostates with inflammatory changes, and lower cancer risk [17, 18].

This report reviews the findings from completed and ongoing clinical trials evaluating the role of MRI in prostate cancer screening and discusses future directions to improve diagnostic accuracy and screening efficiency. The term screening is used to denote the invitation of entire populations, not PSA testing after individual counseling.

## Diagnostic MRI studies

Diagnostic studies have examined how MRI can best be integrated into prostate cancer screening, either as a stand-alone test or within a sequential screening pathway (Table 1a). Most studies define a positive MRI as PI-RADS ≥ 3 and clinically significant prostate cancer as Grade Group ≥ 2. A summary of ISUP Grade Groups [19] alongside the European Association of Urology (EAU) clinical risk categories [13] is provided in Table 2.

**Table 1:** **a** Completed diagnostic MRI trials; **b** Ongoing population-based prostate cancer screening trials using MRI

a									
Trial									
	Number of screened men	Invited ages (years)	PSA cutoff (ng/mL)	PSA density cutoff for biopsy if MRI negative (ng/mL^2^)	MRI protocol	MRI reading	Definition positive MRI	Biopsy protocol	Main endpoint
IP-Prostagram (prospective cohort)	2034 invited by GP, 408 had all tests (years 2018–2019)	50–69							Clinically Significant prostate cancer, GG2–5
PSA			3	-	-			Systematic	
MRI			-	-	bpMRI	Central review	PI-RADS 4–5	Targeted and systematic	
Ultrasound			-	-	-			Targeted and systematic	
Re-Imagine (prospective cohort)	2096 randomly selected from 8 GP surgeries, and invited by letter, 309 eligible responders (years 2019–2022)	50–75	-	≥ 0.12	Axial plane bpMRI			Targeted and systematic	Clinically Significant prostate cancer, GG2–5
MRI	303				Axial T2W and DWI (one b-value 2000 s/mm^2^)	Study radiologists	Likert positive according to study criteria		
VISIONING (prospective cohort)	269 men enrolled at a single center after invitation by GP or urologist (years 2019–2022)	≥ 50 (45 if family history positive)	-	≥ 0.15				Targeted and systematic	Clinically Significant prostate cancer, GG2–5
MRI	229				bpMRI	Central review	PI-RADS 4–5 or PI-RADS ≥ 3 at follow-up of PI-RADS 3 with mpMRI		
MVP (Randomized trial)	Single tertiary center, invitation by ads.1188 assessed for eligibility.	≥ 50							Prostate cancer, GG1–5
PSA	266 allocated		2.6	-				Systematic	
MRI	259 allocated			-	bpMRI	Study radiologist	PI-RADS 4–5	Targeted and systematic	
PROSA (Randomized trial)	Employees and volunteers at a single hospital. 816 assessed for eligibility	49–69		-					Clinically Significant prostate cancer, GG2–5
MRI	374 allocated		-		bpMRI	Study radiologists	PI-RADS 3–5	Targeted	
PSA ± MRI	385 allocated		3 (2.5 if family history positive)		bpMRI	Study radiologists	PI-RADS 3–5	Targeted	

b									
	Number of randomized men	Start ages (years)	PSA cutoff (ng/mL)	Ancillary test	MRI protocol	MRI reading	Definition positive MRI	Biopsy protocol	Main endpoint
Gothenburg 2		50–60							Clinically insignificant prostate cancer, GG1
Screening group	39,000		3 (arms 1&2) or 1.8 (arm 3)	-	mpMRI (bpMRI round 2 and onward)	Study radiologists in consensus	PI-RADS 3–5	Targeted and systematic	
Control group	19,000		Clinical routine	-	Clinical routine	Clinical routine		Clinical routine	
Proscreen		50–63		4KScore					Prostate cancer mortality after 10 and 15 years
Screening group	29,000		3	7.5%	mpMRI	Local radiologists	PI-RADS 3–5	Targeted and systematic	
Control group	88,000		Clinical routine	-	Clinical routine	Clinical routine		Clinical routine	
PROBASE									Metastasized prostate cancer at age 60 years
Early screening	23,000	45	3	-	mpMRI	Local radiologists	PI-RADS 3–5	Targeted and systematic	
Delayed screening	23,000	50	3	-	mpMRI	Local radiologists		Targeted and systematic	
STHLM-3 MRI	12,750 enrolled, 1532 PSA ≥ 3 ng/mL	50–74							Clinically Significant prostate cancer, GG2–5
MRI group	929		3	-	bpMRI	Study radiologists	PI-RADS 3–5	Targeted and systematic	
Systematic biopsy group	603		3	-	-	-		Systematic	
Stockholm 3 reinvite		61–72	3	-					Clinically Significant prostate cancer, GG2–5
Standard group	5134	Not reinvited							
Experimental group	7609	Reinvited			bpMRI	Study radiologists	PI-RADS 3–5	Targeted and systematic	

*bpMRI* bi-parametric MRI, *GG* grade group, *mpMRI* multi-parametric MRI

**Table 2:** International Society of Urological Pathology (ISUP) Grade Groups (GG) and Gleason score versus European Association of Urology clinical risk categories

ISUP Grade Group	Gleason Score	Description	EAU Clinical Risk Category
1	2–6	Low-grade cancer	Low-risk: GG 1 AND PSA < 10 ng/mL AND cT1–2a*
2	7 (3+4)	Favorable intermediate-grade	Intermediate-risk, Favorable: - GG 2 AND PSA < 10 ng/mL AND cT1–2b - OR GG 1 AND PSA 10–19 ng/mL AND cT1–2b - OR GG 1 AND PSA < 10 ng/mL AND cT2b
3	7 (4+3)	Unfavorable intermediate-grade	Intermediate-risk, Unfavorable: - GG 2 AND PSA 10–19 ng/mL AND cT1–2b - OR GG 3 AND cT1–2b
4	8 (4+4, 3+5, 5+3)	High-grade cancer	High-risk: - GG 4 or 5 - OR PSA ≥ 20 ng/mL - OR cT2c
5	9–10 (4+5, 5+4, 5+5)	Highest-grade cancer	High-risk / Locally advanced: - cT3–4 and/or cN+ - Any GG with locally advanced staging

*Tumor stage based on Digital Rectal Examination (DRE)

Bi-parametric MRI as a first-line screening test has been evaluated in several prospective cohort studies: IP-PROSTAGRAM, RE-IMAGINE, and VISIONING [20–22]. Recruitment was predominantly from primary care settings. Typically, men aged over 50 years without a prior history of prostate cancer were included. Strategies to reduce false-positive findings included the use of experienced radiologists, dichotomization of MRI scores [21], defining a positive MRI as PI-RADS 4 [20], and follow-up of PI-RADS 3 with multi-parametric MRI [22]. All three studies demonstrated the potential of MRI to preferentially detect Grade Group ≥ 2 cancer regardless of PSA level (Table 3), with a substantial proportion detected below the commonly used PSA threshold of 3 ng/mL [21, 22]. However, the number needed to scan to diagnose one significant cancer varied considerably, ranging from 11 to 37.

**Table 3:** Outcomes of upfront MRI studies

	IP-Prostagram	RE-IMAGING	VISIONING	MVP	PROSA (arm A)
MRI (*n*)	408	303	229	246	369
Suspicious MRI (*n*)	43 (11%)	48 (16%)	77 (34%)	25 (10%)	46 (12%)
GG1 (*n*)	5 (1.2%)	2 (0.7%)	8 (3.5%)	4 (1.6%)	9 (2.4%)
GG2+ (*n*)	11 (2.7%)	25 (8.3%)	21 (9.2%)	11 (4.5%)	17 (4.6%)
Ratio GG2/GG1	1.8	12.5	2.6	2.8	1.9
GG2 below PSA 3 ng/mL (*n*)	n/a	15	7	n/a	8
Number needed to scan to detect one GG2	37	12	11	22	22

Upfront imaging has also been compared directly with PSA, or the more widely used strategy of initial PSA testing for risk stratification, with MRI reserved for men with PSA values above a predefined cutoff.

The MVP trial randomized 525 men (median age 67.5 years) to screening with upfront MRI or PSA testing (cutoff 2.6 ng/mL) [23]. In the MRI arm, Grade Group 2–5 cancer was detected in 11 of 259 men (4%), compared with 4 of 266 men (2%) in the PSA arm; however, this difference did not reach statistical significance.

The PROSA trial randomized a selected study population of 816 men aged 49–69 years to either upfront MRI or PSA followed by MRI for PSA values > 3 ng/mL [24]. Their median PSA was 2.1 ng/mL. Grade Group 2–5 cancer was detected in 17 men (4.6%) in the upfront MRI group and 7 men (1.8%) in the PSA-first group. Corresponding proportions of Grade Group 1 cancer were 2.4 and 1.0%. Although upfront MRI was cost-effective in this setting, the results cannot be generalized to invited screening populations with much lower average PSA-levels.

Despite these potential advantages, the long-term feasibility and cost-effectiveness of using imaging as an initial screening strategy remain uncertain [25]. Although MRI can detect Grade Group 2 cancer in a substantial proportion of men with PSA below the common threshold 3 ng/mL, it is not known how many of these need to be detected before PSA rises to above 3 ng/mL for treatment to be successful. Moreover, any benefit must be weighed against the harm of increased overdiagnosis, an inevitable consequence of detecting cancer earlier by screening asymptomatic individuals.

## Ongoing randomized screening trials

Several population-based, randomized screening trials now incorporate MRI into their protocols, all using PSA as the initial test followed by pre-biopsy MRI (Table 1b). They are designed for long-term screening with multiple rounds until a specified stop age. However, important characteristics differ, including PSA cutoff, the use of ancillary blood tests, MRI protocols and reading, biopsy thresholds, and biopsy techniques.

The Swedish Gothenburg 2 screening trial has randomized 58,000 men aged 50–60 years to screening or control group [26]. The primary objective is to determine whether replacing standard systematic biopsies in men with PSA ≥ 3 ng/mL with pre-biopsy MRI and MRI-targeted biopsies reduces the detection of low-grade cancer (Grade Group 1) while preserving the detection of clinically significant cancer.

**Fig. 1:**
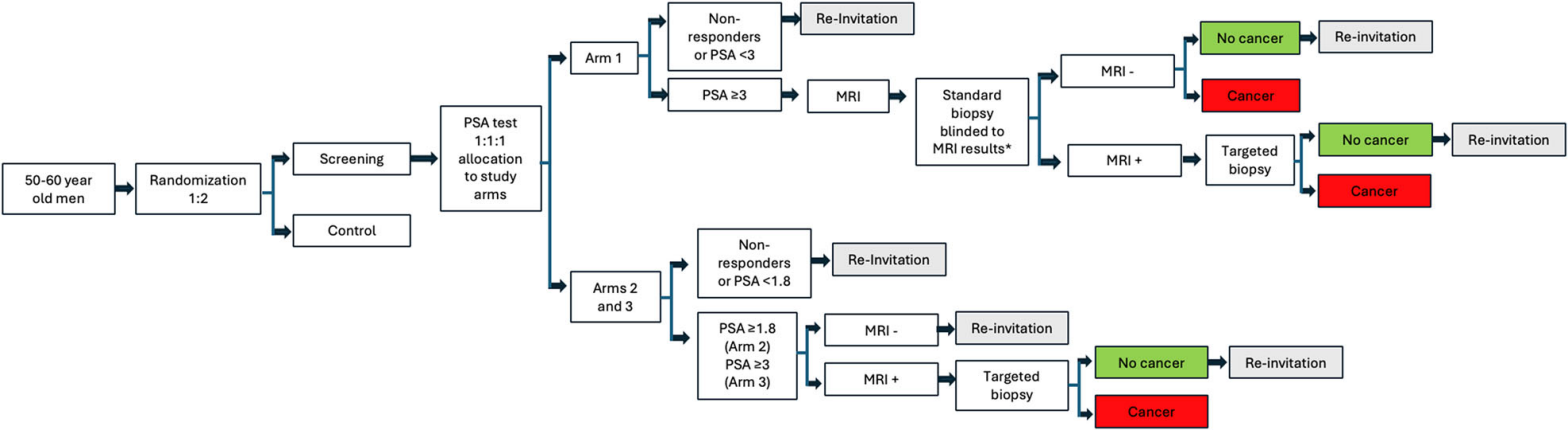
Simplified flowchart of the Gothenburg 2 trial. Men are randomized before consent to either a screening group or a control group. Those in the screening group receive a mailed invitation for PSA testing. Men who choose to participate are then further randomized into one of three study arms, all incorporating MRI. In the reference arm, a systematic biopsy is done regardless of MRI findings*, with an additional targeted biopsy if suspicious lesions are identified. In the experimental arms, only men with suspicious MRI findings undergo biopsy, and only targeted biopsies are taken. Positive MRI is defined as PI-RADS 3–5. Targeted biopsies include 3–4 cores per lesion. The re-invitation interval is 2 years if PSA > 1.2 ng/mL. *Systematic biopsies removed from the study protocol after 1 September 2024 unless PSA > 10 and PSA density > 0.1, or non-diagnostic MRI

The trial protocol illustrates the structure of a long-term prostate cancer screening trial (Fig. 1). Men who are not diagnosed with cancer are reinvited for screening at intervals ranging from 2 to 8 years, depending on their PSA level. The stop age for screening is also PSA dependent, from 62 to 75 years.

In the first screening round, 5994 men were assigned to the reference group (systematic biopsy with additional targeted biopsy when applicable) and 11,986 men to the experimental group (MRI-targeted biopsy only, no systematic biopsy). The participants’ median age was 56 years. Approximately one-third had previously undergone PSA testing, while only 1% had undergone a prior biopsy. Using a PSA cutoff of 3 ng/mL, the relative risk of being diagnosed with Grade Group 1 cancer in the MRI-targeted group compared with the reference group was 0.46 (95% CI, 0.33–0.64). In the reference group, ten additional Grade Group 2 cancers were detected through systematic biopsies in MRI-negative men. These findings indicate that relying solely on MRI-targeted biopsy reduces overdiagnosis of low-risk prostate cancer by about half, at the expense of delaying the diagnosis of a small number of intermediate-grade cancers. [27].

In a 4-year follow-up of the trial, which included both screen-detected cancers and interval cancers, the detection of Grade Group 1 cancer was further reduced in the MRI-targeted biopsy group compared with the systematic biopsy group: the relative risk was 0.49 (95% CI, 0.33–0.71) in the first screening round and 0.25 (95% CI, 0.15–0.42) in round two and beyond [28]. Detection of Grade Groups 2–5 cancer was slightly, although not statistically significantly, lower in the MRI-targeted group (overall relative risk 0.84, 95% CI 0.66–1.07). Only a small number of Grade Group 4–5 or advanced cancers were diagnosed — 15 in the MRI-targeted group and 23 in the systematic biopsy group—and just one of these occurred in a man with a negative MRI.

Proscreen is a population-based screening trial randomizing 117,200 men aged 50 to 63 years to a screening or a control group, recruited from two regions in Finland. Men in the control group are handled according to the standard of care. The main endpoint is prostate cancer mortality after 10 and 15 years of follow-up.

Men randomized to screening are invited to sequential testing with PSA, an ancillary blood test (the 4Kscore), and pre-biopsy MRI. It is estimated that among 1000 screened men, approximately 100 will have PSA ≥ 3 ng/mL, of these 70 will have a 4Kscore above 7.5% and proceed to MRI, 35 will have a positive MRI and undergo targeted biopsy, and 17 will be diagnosed with clinically significant prostate cancer (Grade Group 2–5).

Results from the first screening round have been published, including 15,201 invited men in the screening group and 45,544 men in the control group [29]. After a mean follow-up of 3.2 years, the cumulative incidence of Grade Group 2–5 and Grade Group 1 prostate cancer in the screening group was 1.13% and 0.26%, compared with 0.62% and 0.14% in the control group. These differences correspond to a risk difference of 0.51% (95% CI, 0.33%–0.70%) for detecting clinically significant prostate cancer and 0.11% (95% CI, 0.03%–0.20%) for detecting insignificant prostate cancer. In practical terms, the screening protocol identified one additional clinically significant cancer for every 196 men screened, and one additional clinically insignificant cancer for every 909 men screened. The authors emphasized that these findings are preliminary and should be interpreted cautiously until prostate cancer mortality outcomes become available. In addition, the cost and performance of ancillary tests in repeated screening are yet to be evaluated.

The German PROBASE trial has randomized 50,000 men to screening starting at either age 45 or age 50 years. Participants undergo PSA testing, with MRI and biopsy recommended for all with PSA ≥ 3 ng/mL. Men with PSA < 1.5 ng/mL are reinvited for testing every 5 years, and those with PSA 1.5–2.9 ng/mL every 2 years. The primary objective is to determine whether delayed screening is non-inferior in terms of metastatic prostate cancer at age 60 years. Secondary objectives include evaluating the role of prostate MRI and establishing a biobank.

To date, results have been published for the arm invited to screening at age 45 [17, 30]. Among the 23,411 screened men, only 33 (0.14%) were diagnosed with clinically significant prostate cancer defined as Grade Group ≥ 2. The rate of indeterminate (PI-RADS 3) MRI findings was as high as 43% on local reading, out of which more than half were false positives. On central review, the proportion of PI-RADS 3 lesions was even greater (48%). The specificity for a biopsy threshold of PI-RADS ≥ 3 was only 28%, compared with 88% when the cutoff was raised to PI-RADS ≥ 4. These results highlight the challenges of interpreting prostate MRI in younger men, who tend to have small, denser prostates with inflammatory changes, and suggest that adjustments of both PI-RADS criteria and biopsy thresholds may be necessary in a screening context.

The Swedish STHLM3-MRI trial explored a sequential diagnostic algorithm starting with PSA [31]. Men aged 50 to 74 years with PSA above 3 ng/mL were randomized to either a standard systematic biopsy (*n* = 623) or bi-parametric MRI with a biopsy only if suspicious findings were present (*n* = 929). Grade Group 2–5 cancer was detected in 192 (21%) men in the MRI group and in 106 (18%) in the standard biopsy group, a 3% difference (95% CI −7 to 7). The MRI group also had fewer Grade Group 1 cancers: 4% vs. 12% and half as many biopsy procedures, 32% vs. 73%. The authors concluded that the MRI-targeting biopsy strategy was non-inferior to systematic biopsy for detecting clinically significant prostate cancer.

The STHLM-3MRI REINVITE trial investigates repeated screening with PSA and bi-parametric MRI. Out of 1500 men eligible for rescreening after 2–3 years, 617 (41%) had an MRI, 84 (5.6%) had a PI-RADS 3–5 lesion, and 48 (3.2%) were diagnosed with Grade Group ≥ 2 cancer [32].

The TRANSFORM trial (ISRCTN13801649) is a large-scale UK screening initiative that plans to enroll approximately half a million men in its main phase. The target population is men aged 45 to 75 years with no history of prostate cancer. The initial pilot phase, which started in November 2025, evaluates four different screening strategies, all incorporating pre-biopsy MRI.

The first trial arm uses a PSA cutoff of 3 ng/mL, followed by multi-parametric MRI and both targeted and systematic biopsies. The other three use an abbreviated bi-parametric MRI (“Prostagram”) with targeted biopsies only. Arm 2 applies a pre-MRI PSA cutoff of 1 ng/mL, arm 3 evaluates MRI as the initial screening test, and arm 4 selects men with a polygenic risk score (PRS) over 3.5% for a primary MRI.

The main stage of the trial will adopt the most promising strategy or combination of strategies identified during the pilot phase and assess both its intermediate diagnostic and long-term (10-year) prostate cancer mortality outcomes.

## Knowledge gaps and future developments

Although MRI is now an established part of contemporary prostate cancer screening strategies, several important knowledge gaps and areas for future development must be addressed to ensure effective, scalable, and evidence-based implementation.

### Optimizing MRI protocols and reading for screening

Bi-parametric MRI offers clear advantages in population-based screening by enabling non-invasive imaging and shorter examination times while preserving diagnostic accuracy [20–24, 31, 33–36]. Because diffusion-weighted imaging (DWI) is prone to artefacts, ensuring adequate diagnostic image quality is essential. The updated PI-QUAL guideline now includes assessment of bi-parametric MRI, and its use should be encouraged in screening settings [37].

Accelerating image acquisition using artificial intelligence–based algorithms is a recent development with the potential to yield substantial time savings [38, 39]. Further streamlining may be achieved through fast protocols limited to single-plane imaging [40, 41]. Acquisition of a single very high b-value (b = 2000 s/mm²) without an apparent diffusion coefficient (ADC) map has also shown promising results [21]. However, ongoing screening trials currently adhere to multi-plane, multi b-value PI-RADS–compliant protocols [16, 17, 27, 29, 31].

Reader expertise is integral to reduce variability in MRI assessment. Most screening trials rely on dedicated radiologists and/or centralized reading (Table 1a, b). In locally read organized prostate cancer testing, central review of bi-parametric MRI increased the positive predictive value for detecting Grade Group 2–5 cancer and reduced inter-regional variation in diagnostic outcomes [15]. Structured training programs for radiologists to ensure access to sufficient expertise across healthcare systems should therefore be considered a high priority [42].

Future updates to PI-RADS are anticipated to incorporate bi-parametric MRI and differentiated assessment strategies based on population risk. In addition, the use of PSA density to guide biopsy decisions in men with PI-RADS 3 lesions may reduce unnecessary biopsies [13, 43].

### Use of repeated MRI

The use of MRI in repeated screening rounds is a critical issue, as repeat imaging in previously MRI-negative men detects few clinically important cancers yet substantially increases MRI demand. In the Gothenburg 2 trial, 9 out of 10 men with a previous negative MRI remained MRI negative at the 2-year follow-up [44]. Similarly, in STHLM3 reinvite, only 10 out of 386 (2.6%) men with a previously negative MRI had a PI-RADS 4 or greater after 2–3 years [32]. Better models based on clinical data, in particular PSA density, and imaging features are needed to stratify follow-up intervals. It is possible that men with an initial, unsuspicious MRI and a low PSA density do not require any repeat MRI until their PSA density increases.

### Use of detection-AI in MRI screening

Artificial intelligence (AI) for image interpretation has the potential to greatly improve the efficiency of MRI-based screening programs. For example, AI could help rule out significant findings—particularly relevant given that typically more than two-thirds of screening MRI scans are negative. However, the ethical and medico-legal aspects of fully automated use are unresolved. Less controversially, AI may serve as a second reader to reduce inter-reader variability and enhance diagnostic performance [45]. However, currently available CE-marked AI systems have largely been trained on images from clinical settings with high prevalences of cancer and benign prostatic enlargement. This means that they may not be well-suited for screening settings with younger, mostly asymptomatic men, where inflammatory changes are common and the tolerance for false positives is much lower [46]. In recent work, an AI system trained on bi-parametric screening images achieved lower specificity than expert radiologists [47]. These findings underscore the need for further development and validation of AI systems specifically designed for screening.

### Organized prostate cancer testing (OPT)

**Fig. 2:**
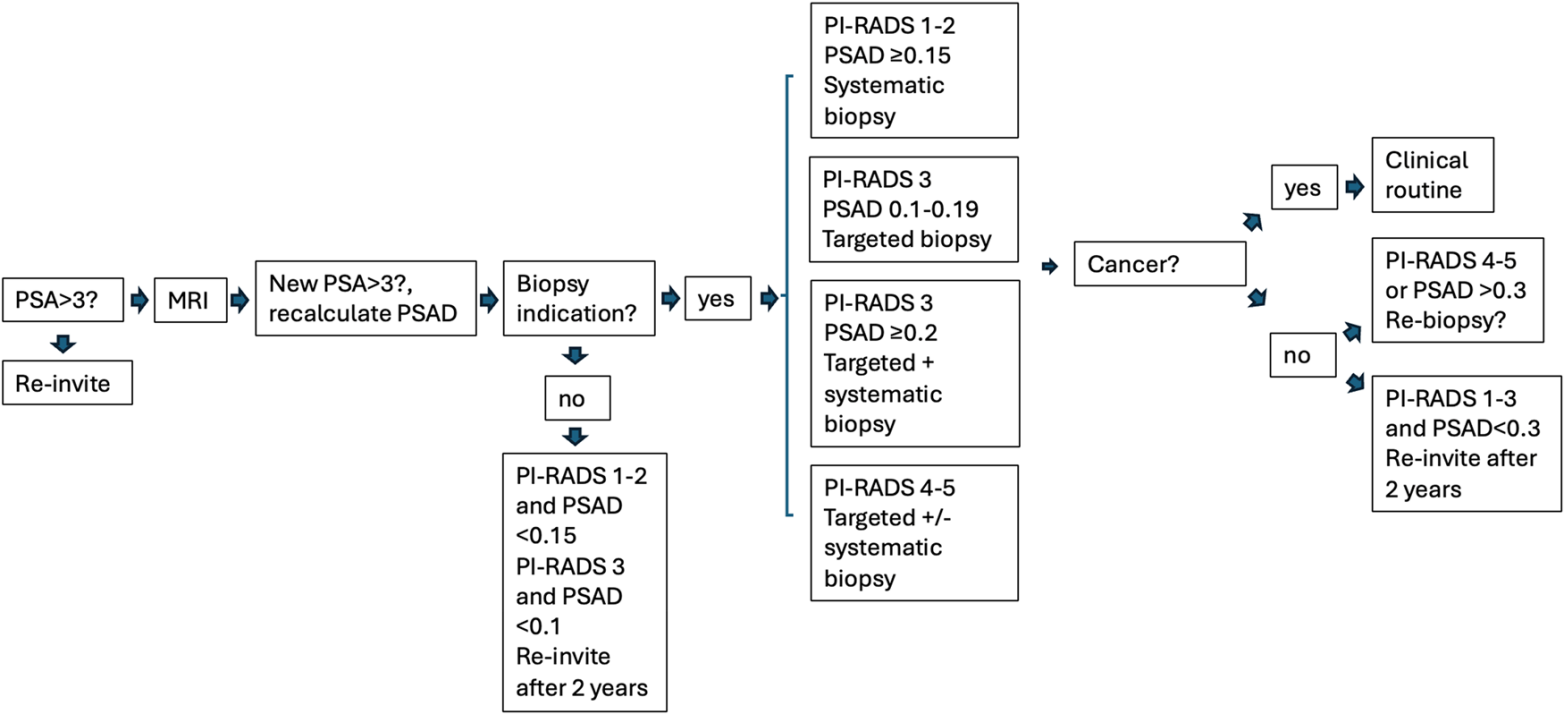
Simplified version of the standard algorithm in Swedish organized prostate cancer testing (OPT). Biopsy indications are based on MRI findings together with PSA density. Men not diagnosed with prostate cancer after MRI ± biopsy are reinvited for PSA testing after 2 years

Finally, the real-world performance and feasibility of MRI-based screening algorithms must be evaluated. European [48] and national [49] initiatives for OPT are underway, providing important insights for future screening programs. In Sweden, regional OPT programs with strictly defined testing and diagnostic algorithms are implemented within public healthcare [50]. A simplified version of the OPT standard algorithm is shown in Fig. 2. In brief, men are invited for PSA testing, and those with PSA ≥ 3 ng/mL undergo pre-biopsy MRI. Biopsy indications are based on MRI findings together with PSA density. Men not diagnosed with prostate cancer after MRI ± biopsy are reinvited for PSA testing after 2 years. Data are collected in the SweOPT register, and key performance indicators are published annually on cancercentrum.se.

**Fig. 3:**
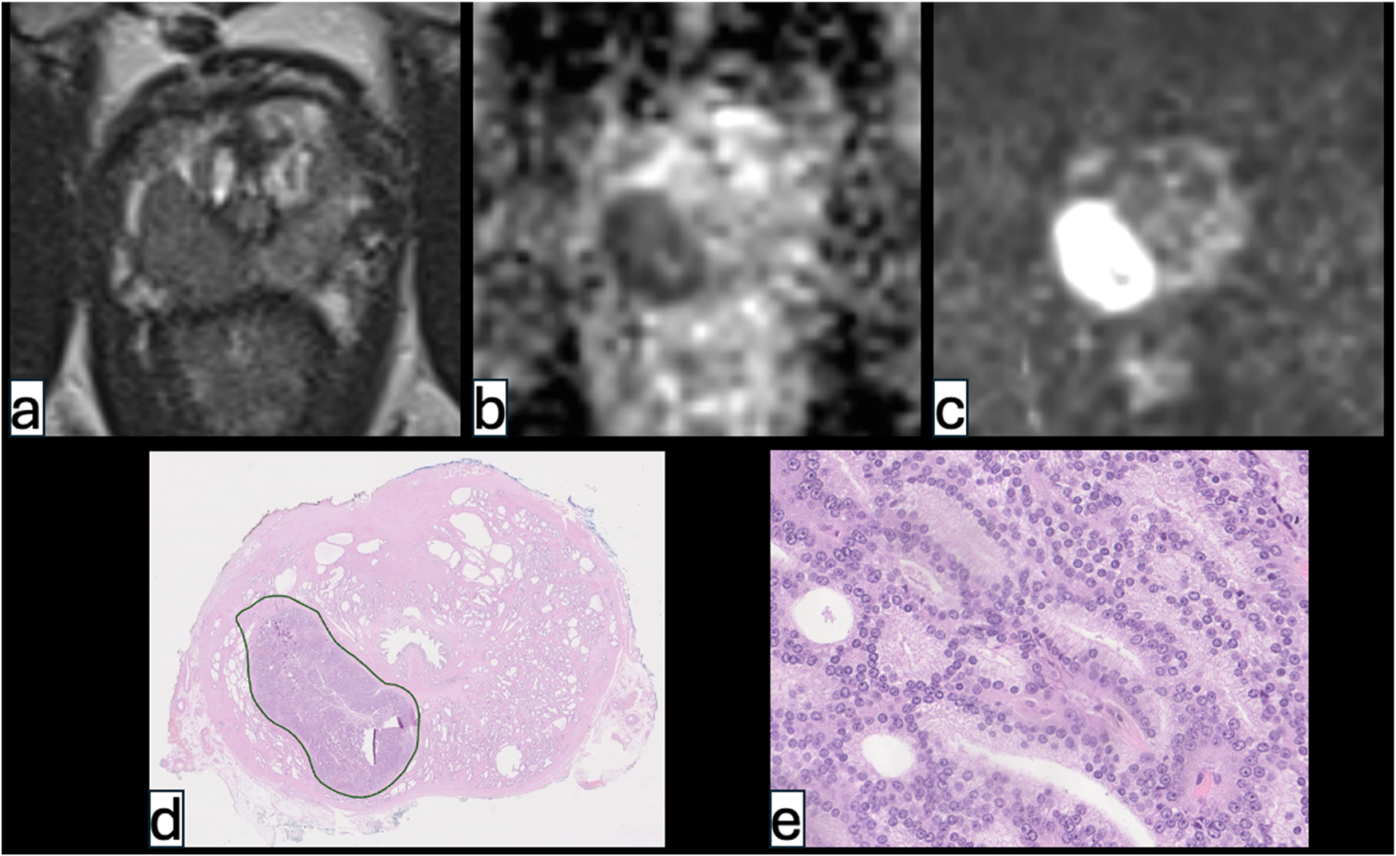
A 50-year-old man participating in Swedish organized prostate cancer testing. PSA 5.6 ng/mL, PSA density 0.24 ng/mL^2^. Bi-parametric MRI with axial T2-weighted imaging (**a**), ADC map (**b**), and high b-value DWI (b = 1500 s/mm²) (**c**) demonstrates a PI-RADS 5 lesion in the dorsal right peripheral zone. MRI-targeted biopsies and robot-assisted laparoscopic prostatectomy (RALP) (**d**), with magnified histopathology (**e**) showing a Grade Group 3 prostate cancer

In the first invitation round targeting all 50-year-old men in Sweden’s three most populous regions, 23,855 of 68,060 men (35%) participated; 699 (2.9%) had PSA ≥ 3 ng/mL. Out of 645 men who had an MRI, 236 (37%) had a suspicious lesion (PI-RADS 3–5), 221 (34%) underwent biopsy, and 93 (14%) were diagnosed with Grade Group 2–5 cancer (Fig. 3). In conclusion, the use of MRI and PSA density biopsy avoided biopsy in almost two-thirds of men. Compliance with the diagnostic pathway was high, although inter-regional variation in diagnostic outcomes revealed a need for further standardization across program components [51].

## Conclusions

In screening, pre-biopsy prostate MRI reduces overdiagnosis and unnecessary biopsies while maintaining detection of clinically significant cancer.

Although MRI has become central in modern prostate cancer screening, key challenges remain—optimizing protocols, managing false positives, and ensuring consistent interpretation. Radiologists are essential to diagnostic quality, and strategies such as centralized reading and AI support may enhance program scalability.

Ongoing trials and organized screening initiatives will help fill knowledge gaps and guide implementation. With continued refinement, MRI-based pathways offer a more effective approach to prostate cancer screening.

## Abbreviations

AI: Artificial intelligence
OPT: organized prostate cancer testing
PSA: prostate–specific antigen
